# Supplementary material for: Characteristics and Expression Analysis of the MYB-Related Subfamily Gene in Rosa chinensis
Source: Int J Mol Sci. 2024 Nov 29;25(23):12854. doi: 10.3390/ijms252312854 (PMC11640956; doi:10.3390/ijms252312854)
Supplement: Supplementary file 1 [file ijms-25-12854-s001.zip › ijms-3320395-supplementary.pdf]

Table S1

Gene_Primer_list

Gene_name	F	R
RcMYB002	CACAGACTCCCACCGACCTCTC	GTTTGCCCACATTTCTCCTCTTGC
RcMYB003	CCTTTAGTCCTGCTGCTGTTGGG	CGTGTATGGTTTCCTCACCTTGGG
RcMYB011	ACAAGGGAGTGGGATGGAGTGAG	TGACTGGCGACCTGGGTTGG
RcMYB020	AATGCTGGTTGCCGTACATCGTC	CTCGCCACTTTCCCTTGCTCTG
RcMYB021	AATGCTGGTTGCCGTACATCGTC	CTCGCCACTTTCCCTTGCTCTG
RcMYB079	CGTCCAAAGAAGCAGGCAATTCG	TCGCCCTTTCATTGTCAGACTTGG
RcActin	AGCGTGGCTATTCCTTCAC	GTACTTCTGGGCAACGGAA
